# Added value of systemic inflammation markers in predicting pulmonary infection in stroke patients: A retrospective study by machine learning analysis

**DOI:** 10.1097/MD.0000000000028439

**Published:** 2021-12-30

**Authors:** Lv Zheng, Lv Wen, Wang Lei, Zhang Ning

**Affiliations:** a Department of Rehabilitation, Shenzhen Longgang Central Hospital, Shenzhen, China; b Department of Rehabilitation, First Affiliated Hospital of Heilongjiang University of Chinese medicine, Harbin, China.

**Keywords:** ensemble analysis, machine learning algorithms, predictive model, pulmonary infection, stroke

## Abstract

Exploring candidate markers to predict the clinical outcomes of pulmonary infection in stroke patients have a high unmet need. This study aimed to develop machine learning (ML)-based predictive models for pulmonary infection.

Between January 2008 and April 2021, a retrospective analysis of 1397 stroke patients who had CT angiography from skull to diaphragm (including CT of the chest) within 24 hours of symptom onset. A total of 21 variables were included, and the prediction model of pulmonary infection was established by multiple ML-based algorithms. Risk factors for pulmonary infection were determined by the feature selection method. Area under the curve (AUC) and decision curve analysis were used to determine the model with the best resolution and to assess the net clinical benefits associated with the use of predictive models, respectively.

A total of 889 cases were included in this study as a training group, while 508 cases were as a validation group. The feature selection indicated the top 6 predictors were procalcitonin, C-reactive protein, soluble interleukin-2 receptor, consciousness disorder, dysphagia, and invasive procedure. The AUCs of the 5 models ranged from 0.78 to 0.87 in the training cohort. When the ML-based models were applied to the validation set, the results also remained reconcilable, and the AUC was between 0.891 and 0.804. The decision curve analysis also showed performed better than positive line and negative line, indicating the favorable predictive performance and clinical values of the models.

By incorporating clinical characteristics and systemic inflammation markers, it is feasible to develop ML-based models for the presence and consequences of signs of pulmonary infection in stroke patients, and the use of the model may be greatly beneficial to clinicians in risk stratification and management decisions.

## 1 Introduction

Stroke is a kind of acute cerebrovascular accident, mainly caused by cerebral vascular rupture of vascular obstruction, which can be manifested as numbness of limbs, transient loss of consciousness, vertigo, etc.[^1^,^2^] Patients with acute stroke may have different degrees of dysphagia, while patients with aspiration diseases are more prone to pneumonia.^[3]^ Clinically, stroke patients mostly occur in middle-aged and elderly people, because the physiological functions of various tissues and organs of the body have declined, so the anti-infection ability is barely satisfactory, and it is easy to complicate with pulmonary infection, which leads to high morbidity and mortality.[^4^,^5^] So far, in randomized clinical trials, prophylactic antibiotic therapy initiated early after stroke did not reduce the risk of pneumonia or death, nor did it lead to better functional outcomes.^[6]^

The exact duration of the incubation period of pneumonia after stroke is unclear. The moment of clinical diagnosis of poststroke pneumonia is defined as the beginning of antibiotics, however, two-thirds of the patients with imaging signs of pulmonary infection did not have clinically significant pneumonia.[^7^,^8^] Herein, understanding the patterns of stroke, especially risk factors for pulmonary infection in stroke patients, is vitally crucial to improving diagnosis, treatment, and health education for patients. The current conventional statistical methods, such as logistic regression, have been used for the prediction of pulmonary infection in stroke patients.^[2]^ Machine learning (ML) is the scientific discipline that focuses on how computers learn from data, which seeks to learn relationships from data, and computer science, with its emphasis on efficient computing algorithms.[^9^,^10^,^11^] Many prediction models based on ML algorithms have been used in clinical diagnosis and prognosis evaluation.[^12^,^13^] However, the supervised ML algorithm has not been used to predict the risk of pulmonary infection in stroke patients. Therefore, there is an urgent need for new and reliable methods to predict pulmonary infection in stroke patients.

It was reported that inflammation plays an important role in the pathogenesis of ischemic stroke and other forms of ischemic brain injury.^[14]^ The inflammatory factor may play a key role in the pathogenesis of pulmonary inflammatory diseases including pulmonary infection, pulmonary fibrosis, lung injury.^[15]^ However, few reports have incorporated clinical characteristics and inflammatory features as in our study. Our results indicate that the levels of inflammatory factors in stroke patients with pulmonary infection are significantly increased, which may be a potential candidate predictor.

This study aimed to develop and validate effective ML-based models incorporating relevant inflammatory and clinical variables for the individual prediction of pulmonary infection in stroke patients. In addition, we also compared the prediction performance of the ML-based prediction model with the traditional prediction model from the aspects of model recognition ability, model fitting performance, and clinical application.

## 2 Materials and methods

### 2.1 Data source and study design

**Figure 1:**
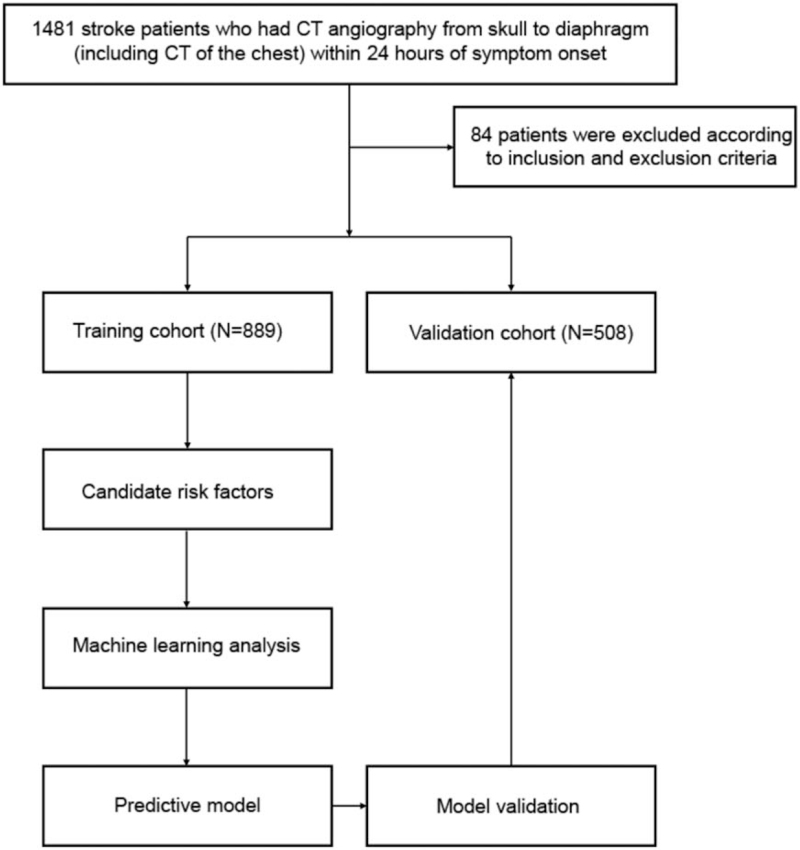
The flow chart of patient selection and data process.

Between January 2008 and April 2021, we retrospectively collected data on 1397 patients with stroke at the Department of Rehabilitation, Shenzhen Longgang Central Hospital, which was included in the cohort for the development of the ML-based predictive models (Internal cohort). The inclusion criteria were as follows: all patients met the diagnostic criteria of stroke; all patients were in acute attack stage; and all patients had complete follow-up data. The exclusion criteria were as follows: patients had a pulmonary infection before admission; diagnosed with immune system or blood system diseases; severe liver and kidney insufficiency or malignant tumor existed; and the clinical data were incomplete. The study was approved by the Institutional Ethics Committee of the Shenzhen Longgang Central Hospital, and written informed consent was obtained from all patients. In addition, eligible patients who were diagnosed with stroke between April 6, 2014 and March 31, 2021 at the Department of Rehabilitation, First Affiliated Hospital of Heilongjiang University of Chinese medicine, were entered into the external validation cohort. The study flow diagram of the selection process was summarized in Figure 1.

### 2.2 Data collection and outcomes

We extracted medical data for demographic factors and clinicopathological parameters, including age, gender, body mass index, comorbidities (hypertension, diabetes, hyperlipidemia, coronary heart disease, etc), types of stroke (cerebral ischemia or hemorrhage), invasive procedure (endotracheal intubation, tracheotomy, indwelling gastric tube, sputum suction, etc), time in ambulation, dysphagia (eating assessment tool-10) and disturbance of consciousness (according to the Coma Recovery Scale-Revised score).^[16]^ We also collected routine laboratory measurements including white blood cell count (WBC, 10^9^/L), neutrophil count (10^9^/L), lymphocyte count (10^9^/L), platelet count (10^9^/L), monocyte count (10^9^/L), C-reactive protein (CRP), and procalcitonin (PCT) were also used to assess the risk of pulmonary infection after stroke.[^17^,^18^] Within 24 hours after admission, fasting venous blood was collected, and serum was taken for enzyme-linked immunosorbent assay to monitor relevant indicators. The outcome of this study was a pulmonary infection, which was divided into infection group and noninfection group according to whether pulmonary infection occurred during hospitalization.^[1]^

### 2.3 Construction of ML-based models

All patients from the internal cohort were randomly divided into training and testing groups at a ratio of 7:3, keeping the distribution of infection group and noninfection data in both groups consistent. Five algorithms were applied to predict pulmonary infection, including generalized logistic regression (GLR), random forest classifier (RFC), artificial neural network (ANN), decision tree, and extreme gradient boosting.[^19^,^20^,^21^,^22^] Among all 5 algorithms, GLR was considered conventional methods, and the others are representative supervised ML-based algorithms. In addition, ten-fold cross-validation and out-of-bag methods were used to test the accuracy of a machine learning algorithm. Decision curve analysis (DCA) is a method to evaluate prediction models and diagnostic tests.^[23]^ Therefore, the prediction ability of these models was evaluated by the DCA and receiver operating characteristic (ROC) curve.

### 2.4 Statistical analysis

Descriptive analysis calculated the intermediary and quartile range of continuous variables and expressed the frequency and proportion of categorical variables. Pearson chi-square test or Fisher exact test was used for categorical variables, and Mann–Whitney *U* test was used for continuous variables. As for the GLR model, we use stepwise regression to determine the final model. The Akaike information standard and concordance index analysis was used to evaluate the model fitting performance.^[24]^ For the internal verification method, we take the method of bootstrap resampling. To obtain the best cutoff value of candidate variables, we use the Youden index in the ROC curve to evaluate the performance. All analysis was performed using the Python programming language (version 3.9.2, Python Software Foundation, https://www.python.org/) and R Project for Statistical Computing (version 4.0.4, http://www.r-project.org/). All tests were two-sided, and *P* < .05 was considered statistically significant.

## 3 Results

### 3.1 Patient characteristics

Initially, we identified 1481 eligible patients with stroke. After screening, 1397 patients were finally included in this study, consisting of 389 (27.85%) patients who had a pulmonary infection. Briefly, 244 (27.45%) and 145 (28.54%) patients had a pulmonary infection in the internal and external cohorts, respectively. Compared to patients without pulmonary infection, those who suffered pulmonary infection were more likely to accompany with abnormal inflammatory factor ratio, basic diseases, disturbance of consciousness, dysphagia, and invasive operation. The clinical characteristics for the training and external validation cohorts were summarized in Table 1. Collectively, combined with basic diseases were the most prevalent comorbidity, disturbance of consciousness, and dysphagia remained the most common symptom.

**Table 1:** Clinical and serological characteristics of stroke patients with or without pulmonary infection.

	Training set			Validation set			
Variables	Overall (N = 889)	Noninfection (N = 645)	Infection (N = 244)	Overall (N = 508)	Noninfection (N = 363)	Infection (N = 145)	*P*-value
Age (median [IQR])	58.00 [47.00, 68.00]	58.00 [47.00, 69.00]	57.00 [47.00, 65.25]	58.00 [47.00, 67.25]	58.00 [48.00, 68.00]	57.00 [47.00, 65.00]	.21
BMI (median [IQR])	23.00 [21.00, 25.00]	24.00 [22.00, 26.00]	22.00 [20.00, 24.00]	23.00 [21.00, 25.00]	24.00 [22.00, 26.00]	22.00 [20.00, 24.00]	<.01
Sex (%)							
Female	462 (52.0)	348 (54.0)	114 (46.7)	269 (53.0)	200 (55.1)	69 (47.6)	.15
Male	427 (48.0)	297 (46.0)	130 (53.3)	239 (47.0)	163 (44.9)	76 (52.4)	
Smoking (%)							
Yes	444 (49.9)	330 (51.2)	114 (46.7)	272 (53.5)	198 (54.5)	74 (51.0)	
No	445 (50.1)	315 (48.8)	130 (53.3)	236 (46.5)	165 (45.5)	71 (49.0)	.53
Diabetes (%)							
Yes	514 (57.8)	436 (67.6)	78 (32.0)	291 (57.3)	246 (67.8)	45 (31.0)	
No	375 (42.2)	209 (32.4)	166 (68.0)	217 (42.7)	117 (32.2)	100 (69.0)	<.01
Hypertension (%)							
Yes	421 (47.4)	304 (47.1)	117 (48.0)	249 (49.0)	174 (47.9)	75 (51.7)	
No	468 (52.6)	341 (52.9)	127 (52.0)	259 (51.0)	189 (52.1)	70 (48.3)	.51
Coronary heart disease (%)							
Yes	442 (49.7)	316 (49.0)	126 (51.6)	256 (50.4)	185 (51.0)	71 (49.0)	
No	447 (50.3)	329 (51.0)	118 (48.4)	252 (49.6)	178 (49.0)	74 (51.0)	.75
Hyperlipidemia (%)							
Yes	443 (49.8)	318 (49.3)	125 (51.2)	255 (50.2)	192 (52.9)	63 (43.4)	
No	446 (50.2)	327 (50.7)	119 (48.8)	253 (49.8)	171 (47.1)	82 (56.6)	.06
Consciousness disorder (%)							
Yes	304 (34.2)	132 (20.5)	172 (70.5)	194 (38.2)	93 (25.6)	101 (69.7)	
No	585 (65.8)	513 (79.5)	72 (29.5)	314 (61.8)	270 (74.4)	44 (30.3)	<.01
Dysphagia (%)							
Yes	286 (32.2)	87 (13.5)	199 (81.6)	176 (34.6)	60 (16.5)	116 (80.0)	
No	603 (67.8)	558 (86.5)	45 (18.4)	332 (65.4)	303 (83.5)	29 (20.0)	<.01
Invasive procedure (%)							
Yes	356 (40.0)	157 (24.3)	199 (81.6)	214 (42.1)	100 (27.5)	114 (78.6)	
No	533 (60.0)	488 (75.7)	45 (18.4)	294 (57.9)	263 (72.5)	31 (21.4)	<.01
Time to ambulation (%)							
>7 d	308 (34.6)	120 (18.6)	188 (77.0)	168 (33.1)	63 (17.4)	105 (72.4)	
≤7 d	581 (65.4)	525 (81.4)	56 (23.0)	340 (66.9)	300 (82.6)	40 (27.6)	<.01
WBC (median [IQR])	9.89 [8.43, 11.32]	9.12 [8.08, 10.50]	12.23 [10.80, 13.91]	9.95 [8.42, 11.35]	9.19 [8.07, 10.37]	12.39 [10.83, 14.14]	<.01
CRP (median [IQR])	20.18 [16.89, 23.12]	18.68 [16.02, 21.31]	27.19 [22.95, 30.39]	20.44 [17.11, 23.41]	18.82 [16.07, 21.61]	27.53 [23.50, 30.79]	<.01
PCT (median [IQR])	2.12 [1.70, 2.48]	1.89 [1.58, 2.19]	3.44 [2.87, 3.99]	2.13 [1.67, 2.60]	1.88 [1.56, 2.20]	3.49 [2.78, 3.99]	<.01
SIL-2R (median [IQR])	367.00 [321.00, 400.00]	342.00 [308.00, 377.00]	438.50 [394.00, 482.00]	368.00 [320.75, 403.00]	338.00 [308.00, 381.00]	440.00 [407.00, 488.00]	<.01
NC (median [IQR])	3.21 [2.54, 3.87]	2.88 [2.31, 3.47]	4.66 [3.78, 5.27]	3.25 [2.55, 3.89]	2.83 [2.33, 3.48]	4.63 [3.87, 5.27]	<.01
LC (median [IQR])	1.81 [1.35, 2.20]	1.62 [1.20, 2.03]	2.21 [1.82, 2.50]	1.78 [1.34, 2.22]	1.59 [1.19, 2.06]	2.22 [1.79, 2.52]	<.01
PLT (median [IQR])	176.00 [116.00, 232.00]	177.00 [119.00, 237.00]	172.00 [111.50, 225.25]	179.00 [116.75, 237.25]	187.00 [122.50, 244.50]	162.00 [108.00, 218.00]	<.01
PLR (median [IQR])	97.00 [64.80, 138.33]	109.48 [73.26, 152.73]	76.71 [51.16, 102.09]	99.60 [63.38, 142.08]	114.85 [75.99, 163.97]	72.43 [47.62, 101.36]	<.01
NLR (median [IQR])	1.89 [1.45, 2.44]	1.82 [1.35, 2.35]	2.13 [1.70, 2.53]	1.90 [1.44, 2.50]	1.80 [1.36, 2.45]	2.17 [1.67, 2.52]	<.01

### 3.2 Candidate factors selection and ML-based model construction

**Figure 2:**
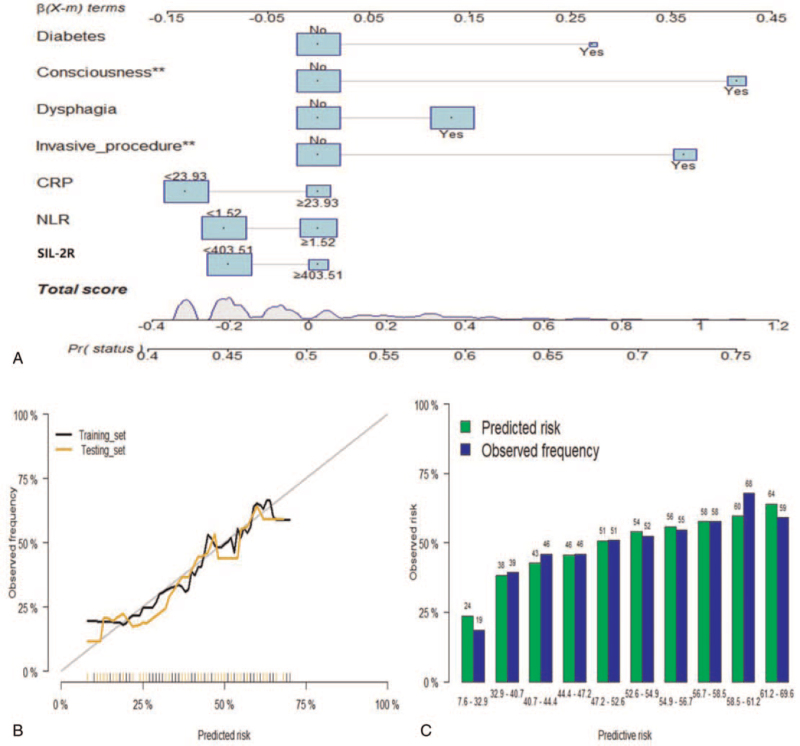
Generalized linear model. A. Nomograms conveying the results of the candidate factors for predicting pulmonary infection. B. Calibration curves for internal validation of the nomogram. C. Predicted risk histogram comparing predicted risk of the nomogram with the observed frequency.

**Figure 3:**
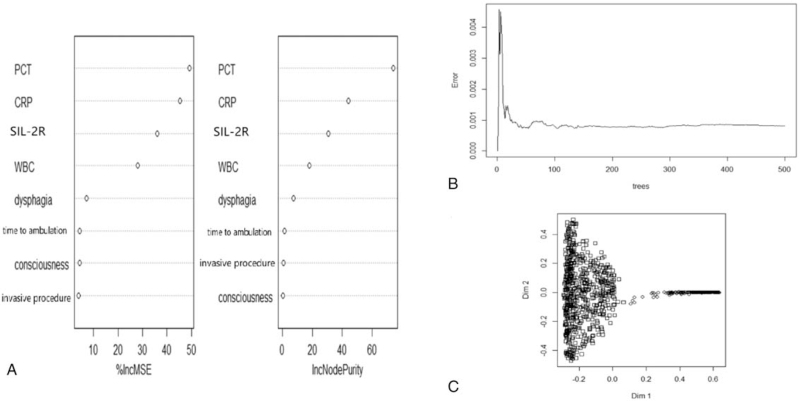
Random forest classifier model. A. The candidate factors associated with pulmonary infection were ordered according to the mean decreased Gini index. B. Relationship of dynamic changes between the prediction error and the number of decision trees. C. Performance of the prediction model with increasing numbers of features in the principal component analysis.

Through feature selection, 21 variables of each algorithm are sorted according to their predictive importance. As shown in Figure 2, the top-rank predictors calculated via GLR were PCT, CRP, soluble interleukin-2 receptor (SIL-2R), WBC, dysphagia, time to ambulation, consciousness disorder, and invasive procedure (Table S1, Supplemental Digital Content). Consistent with the results of RFC (Fig. 3A & Table S2, Supplemental Digital Content), the predictive variables obtained by other ML-based algorithms indicated that inflammatory factors showed robust predictive performance. In addition, the predictive performance of most models reached a plateau when these variables were introduced, while inflammatory factors removed begins to decrease when it reaches the highest point (Fig. 3B). To further verify the prediction efficiency of the model, our results demonstrated that added value of systemic inflammation markers performed a robust discrimination effect (Fig. 3C).

### 3.3 Prediction performance and clinical application of ML-based model

**Figure 4:**
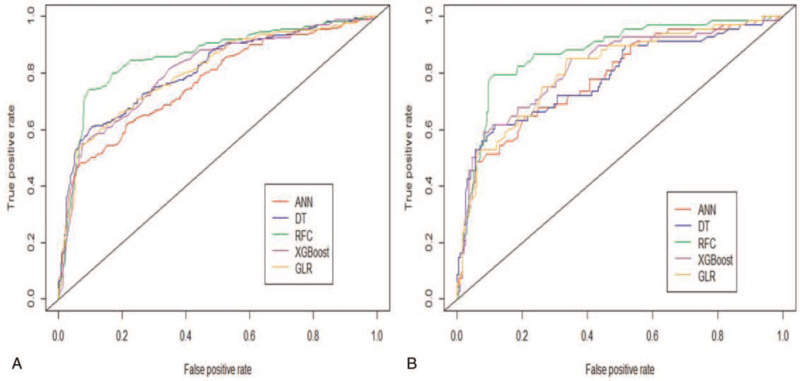
ROC curve analysis compares the prediction efficiency associated with predicting pulmonary infection using machine learning algorithms. A. Internal training set. B. External validation set.

**Figure 5:**
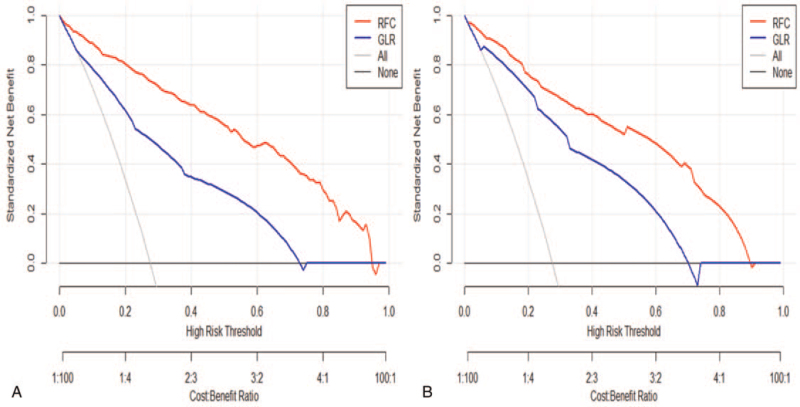
Decision curve analysis compares the net benefits associated with predicting pulmonary infection using RFC and GLR models. A. Internal training set. B. External validation set.

In the training cohort, the predictive performance of all ML-based models was shown in Figure 4. The optimal predictive performance was observed in RFC (area under the curve [AUC] = 0.87, 95%CI: 0.41–1.33), followed by ANN (AUC = 0.78, 95%CI: 0.32–1.24), decision tree (AUC = 0.81, 95%CI: 0.34–1.28), extreme gradient boosting (AUC = 0.82, 95%CI: 0.38–1.26), and GLR (AUC = 0.79, 95%CI: 0.29–1.29). In addition to the ANN model, other ML-based models performed better than the conventional GLR model. Furthermore, the DCA showed the clinical values of these models, consistent with ROC analysis, all of these ML-based predictive models presented better net benefit than the GLR model (Fig. 5). In the validation cohort, the RFC model with top-rank variables achieved ideal predictive performance and had the highest net benefits almost across the entire range of threshold probabilities, which is considered to be the best prediction model compared with other ML-based models, also beyond the GLR model. The AUCs of the 5 models ranged from 0.78 to 0.87. Meanwhile, the DCA also presented a robust performance in the external validation cohort.

## 4 Discussion

Pulmonary infection is one of the most common complications of a stroke, which can aggravate the development of the disease and cause poor prognosis.^[25]^ Therefore, it is very important to accurately identify the patients who are most likely to have a pulmonary infection, which may help clinicians to make decisions and consult. In this population-based study, we not only identified the risk factors of pulmonary infection in stroke patients but also established an ML-based prediction model of pulmonary infection in stroke patients, which filled the gap in this field. To our knowledge, our study provides a potential model for predicting pulmonary infection in stroke patients by combining clinical features and inflammatory markers for the first time.

Increasing evidence has demonstrated decisive roles for inflammatory indexes in the pulmonary infection.[^26^,^27^,^28^] The pneumonia-associated inflammatory state can release a bystander autoimmune response to central nervous system antigens, which leads to a vicious circle.^[28]^ It is speculated that inflammation plays a crucial role in all stages of stroke.^[29]^ Herz et al^[30]^ reported that neutrophils are the main leukocytes that aggravate brain injury during cerebral ischemia. In addition, lymphocytes are also part of the inflammatory response, inhibiting and controlling the worsening inflammatory process, and decreasing in the peripheral blood of stroke patients.^[31]^ Consistent with previous research reports, CRP, WBC, SIL-2R, and NLR are known to be independent contributing factors for stroke development.^[32]^ Similar to NLR, PLR is an easy to obtain and cheap blood test method reflecting platelet reactivity.^[32]^ In this study, we found that the levels of inflammatory factors in stroke patients with pulmonary infection were significantly increased, indicating that there was a significant correlation between inflammatory factors and stroke patients with pulmonary infection.

Using conventional univariate analysis, systemic inflammation markers were found to be associated with pulmonary infection. As a result, the PCT, CRP, SIL-2R, WBC, dysphagia, time to ambulation, consciousness disorder, and invasive procedure were independent risk factors of pulmonary infection in stroke patients. Next, to further explore the interactive relationship between inflammation markers and germ pulmonary infection, we used ML algorithms to identify the candidate variables. It is worth noting that inflammation markers were used to confirm their importance through machine learning feature selection. Similarly, the PCT, CRP, SIL-2R, and WBC were indicated to be the most contributive risk factors of pulmonary infection, which was similar to the results of logistic regression analysis. Cumulatively, this evidence indirectly supported our conclusion that added value of systemic inflammation markers in predicting pulmonary infection in stroke patients. To further verify the predictive effectiveness of adding inflammatory factors as modeling, we found that most of the 5 models maintain high AUC levels, and all ML-based models maintain AUC between 0.78 and 0.87. Compared with the logistic regression model, the iterative model constructed by machine learning has better prediction performance. Collectively, these results verified our hypotheses that the ML-based model is effective in predicting pulmonary infection in stroke patients by combining clinical and inflammatory markers.

DCA has been used in many fields of medical research and has shown great potential in clinical application. Most importantly, it can be directly applied to data sets and does not need the external data about cost, benefit, and preference that traditional decision analysis techniques usually need.^[33]^ As systemic inflammatory response markers are easily obtained and economical in clinical practice. Herein, the combination of inflammatory factors and clinical indicators for integrated learning can obtain efficient, convenient, and economic prediction performance, thus providing a reliable diagnosis and prediction system for stroke patients with pulmonary infection. In addition, we also tested the prediction model through the external validation set, and the results show that the prediction performance is very robust, which indicates that the prediction model has good universality.

There are some limitations to our study. First, since the patients in the training set are completely from a single center, the current retrospective study has exposed it to selection bias. Second, although we use external data validation of a single-center with small sample size, these findings also need further multi-institutional validation with a larger sample size. Third, deep learning algorithms usually find their own rules and do not leave audit clues to explain the decision (black box problem), which is essentially opaque and has not been overcome. Therefore, our results still need to be verified through clinical practice in the future.

## 5 Conclusion

In summary, based on the analysis of the integrated machine learning algorithm, we determined the optimal model for the prediction of stroke pulmonary infection. Based on ROC analysis and DCA evaluation, the ML-based model performed better than the conventional linear regression model, and the RFC model performs best. In addition, the feature selection approach identified that inflammatory factors were the most important predictive risk factors for pulmonary infection.

## Author contributions

**Conceptualization:** Lv Zheng.

**Data curation:** Wang Lei.

**Investigation:** Lv Wen, Zhang Ning.

**Software:** Zhang Ning.

**Supervision:** Lv Zheng.

**Visualization:** Wang Lei.

**Writing – original draft:** Lv Wen, Zhang Ning, Wang Lei.

**Writing – review & editing:** Lv Zheng.

## Supplementary Material

Supplemental Digital Content

## Supplementary Material

Supplemental Digital Content
