# Supplementary material for: Added value of systemic inflammation markers in predicting pulmonary infection in stroke patients: A retrospective study by machine learning analysis
Source: Medicine (Baltimore). 2021 Dec 30;100(52):e28439. doi: 10.1097/MD.0000000000028439 (PMC8718201; doi:10.1097/MD.0000000000028439)
Supplement: Supplemental Digital Content [file medi-100-e28439-s001.docx]

Tables1. The predictive performances of different models associated with pulmonary infection

| GLR model | Multivariate analysis | | Discrimination | | | |
| --- | --- | --- | --- | --- | --- | --- |
|  | OR(95%CI) | P-value | Brier | R^2^ | C-index | AIC |
| **Model1** |  |  |  |  |  |  |
| Diabetes  (Yes vs No) | 2.14(1.61-2.67) | <0.01 |  |  |  |  |
| Consciouness disorder  (Yes vs No) | 2.11(1.68-2.54) | 0.02 |  |  |  |  |
| Dysphagia  (Yes vs No) | 1.35(0.82-1.88) | <0.01 |  |  |  |  |
| Invasive procedure  (Yes vs No) | 2.27(1.76-2.78) | 0.03 | 0.06 | 0.07 | 0.792 | 278.22 |
| CRP  (<23.93 vs ≥23.93) | 1.64(1.13-2.14) | <0.01 |  |  |  |  |
| NLR  (<1.52 vs ≥1.52) | 2.01(1.47-2.55) | 0.03 |  |  |  |  |
| SIL-2R  (<403.51 vs ≥403.51) | 1.57(1.16-1.98) | <0.01 |  |  |  |  |
| **Model2** |  |  |  |  |  |  |
| Diabetes  (Yes vs No) | 2.31(1.92-2.70) | <0.01 |  |  |  |  |
| Consciouness disorder  (Yes vs No) | 1.97(1.54-2.40) | <0.01 |  |  |  |  |
| Dysphagia  (Yes vs No) | 1.62(1.14-2.10) | 0.04 | 0.05 | 0.06 | 0.761 | 301.52 |
| Invasive procedure  (Yes vs No) | 2.28(1.75-2.81) | 0.03 |  |  |  |  |
| NLR  (<1.52 vs ≥1.52) | 1.97(1.50-2.44) | <0.01 |  |  |  |  |
| **Model3** |  |  |  |  |  |  |
| Diabetes  (Yes vs No) | 2.92(2.38-3.46) | <0.01 |  |  |  |  |
| Consciouness disorder  (Yes vs No) | 2.06(1.62-2.50) | <0.05 |  |  |  |  |
| Dysphagia  (Yes vs No) | 1.59(1.13-2.05) | <0.01 | 0.06 | 0.07 | 0.745 | 314.60 |
| Invasive procedure  (Yes vs No) | 2.47(1.92-3.02) | <0.01 |  |  |  |  |
| Time to ambulation  (≤7d vs ＞7d) | 1.56(1.01-2.11) | 0.04 |  |  |  |  |

Notes. *. Continuous variable.

Abbreviations. AIC: Akaike information criterion. GLR model: generalized linear regressionmodel. OR: odds ratio. 95%CI: 95% confidence level.
