# Supplementary material for: Added value of systemic inflammation markers in predicting pulmonary infection in stroke patients: A retrospective study by machine learning analysis
Source: Medicine (Baltimore). 2021 Dec 30;100(52):e28439. doi: 10.1097/MD.0000000000028439 (PMC8718201; doi:10.1097/MD.0000000000028439)
Supplement: Supplemental Digital Content [file medi-100-e28439-s002.docx]

Tables2. The weight of candidate variables associated with pulmonary infection

| Variables | MDA | MDG |
| --- | --- | --- |
| WBC | 0.07 | 17.83 |
| CRP | 0.11 | 44.30 |
| PCT | 0.17 | 74.20 |
| SIL-2R | 0.10 | 30.74 |
| Consciousness disorder | 0.00 | 0.19 |
| Dysphagia | 0.00 | 7.49 |
| Invasive procedure | 0.00 | 0.61 |
| Time to ambulation | 0.00 | 1.37 |

Abbreviations. MDA: Mean Decrease Accuracy. MDG: Mean Decrease Gini.
